# Postoperative prostate‐specific antigen kinetics after holmium laser enucleation of the prostate in men with low‐risk prostate cancer

**DOI:** 10.1002/bco2.70255

**Published:** 2026-08-10

**Authors:** Hasim Bakbak, Gurpremjit Singh, Ahmad Abdelaziz, Juanita Velasquez Ospina, Shlomo Resnik, Connor Stephen Nee, Archan Khandekar, Jonathan E. Katz, Sanoj Punnen, Robert Marcovich, Mark L. Gonzalgo, Dipen J. Parekh, Hemendra Navinchandra Shah

**Affiliations:** ^1^ Desai Sethi Urology Institute University of Miami Miller School of Medicine Miami Florida USA; ^2^ University of Miami Miller School of Medicine Miami Florida USA

**Keywords:** active surveillance, benign prostatic hyperplasia, enlarged prostate, HoLEP, prostate cancer, PSA, screening

## Abstract

**Objectives:**

To characterize postoperative prostate‐specific antigen (PSA) kinetics after holmium laser enucleation of the prostate (HoLEP) in men with low‐risk prostate cancer (PCa) managed with active surveillance and to compare these with men without PCa, establishing a benchmark for expected PSA behaviour after HoLEP in low‐risk disease.

**Patients and Methods:**

We retrospectively reviewed a prospectively maintained HoLEP database at a tertiary centre from 2017 to 2025. Patients were stratified into benign and low‐risk PCa cohorts, with low‐risk PCa defined as Grade Group 1 disease. Patients with clinically significant PCa were excluded. PSA values were assessed at standardized postoperative time points up to 5 years. PSA reduction, PSA velocity (PSAV), PSA doubling time and surveillance outcomes were compared between groups.

**Results:**

A total of 769 patients were included (692 benign, 77 low‐risk PCa). Baseline characteristics were broadly comparable. Both groups demonstrated marked and similar PSA reductions after HoLEP, with no significant difference in percent PSA decline from baseline (91.6% vs. 91.1%, *p* = 0.953) or 3‐month PSA (0.39 vs. 0.40 ng/mL, *p* = 0.858). PSA remained low throughout follow‐up in both cohorts. PSAV was numerically higher in the low‐risk cohort but did not differ significantly (0.127 vs. 0.036 ng/mL/year, *p* = 0.259). PSA doubling time was shorter in the low‐risk cohort, though not significantly (1.74 vs. 2.40 years, *p* = 0.297). A greater proportion of low‐risk patients had PSA exceeding 1.0 ng/mL, though this was not significant (23.4% vs. 17.3%, *p* = 0.249).

**Conclusion:**

HoLEP produces a substantial PSA ‘reset’ with sustained low postoperative PSA in both benign and low‐risk PCa patients. Although low‐risk patients showed numerically higher PSAV, shorter PSA doubling time and greater likelihood of PSA exceeding 1.0 ng/mL, overall postoperative PSA trajectories were statistically comparable between groups. These findings provide a reference for expected PSA kinetics after HoLEP in men with low‐risk PCa undergoing active surveillance.

## 1 INTRODUCTION

Holmium laser enucleation of the prostate (HoLEP) is recommended by guidelines as a size‐independent surgical option for the management of benign prostatic obstruction (BPO), with durable and effective relief.^1^, ^2^, ^3^ Following HoLEP, serum prostate‐specific antigen (PSA) typically declines by more than 80%, establishing a new postoperative PSA nadir.^4^, ^5^, ^6^ Men with low‐risk prostate cancer (PCa) on active surveillance who undergo HoLEP for BPO may remain on surveillance after surgery through shared decision‐making.^7^ In addition, 5.6% to 23.3% of patients undergoing HoLEP for presumed BPO are found to have incidental PCa (iPCa) on histopathologic examination of the enucleated tissue.^8^, ^9^ Most of these cases are low‐risk Grade Group 1 (GG1) disease and are managed with active surveillance (AS).^8^, ^9^, ^10^ However, AS is not always indefinite, as nearly 48% of men initially diagnosed with low‐risk PCa proceed to definitive treatment within 10 years.^11^

In this context, PSA kinetics after HoLEP may help both establish an expected postoperative nadir and inform surveillance in men with known or incidentally diagnosed low‐risk PCa.^4^, ^12^, ^13^ PSA velocity (PSAV) after surgery for BPO may provide additional oncologic context, as higher postoperative PSAV has been reported in men with cancer compared with those with benign pathology.^14^, ^15^ Although guidelines do not support use of PSAV alone for risk stratification, prior studies suggest it may serve as a complementary marker of progression in men with low‐risk PCa managed with AS.^7^, ^16^ Most existing studies on PSA kinetics have focused on predicting clinically significant PCa (csPCa).^14^, ^17^, ^18^ However, currently available PSA thresholds and risk tools were developed in surgery‐naive populations and may not be applicable after HoLEP, leaving PSA interpretation in this setting poorly defined.^11^

Given this background, a clearer understanding of PSA kinetics after HoLEP in men with GG1 disease managed with AS is needed. Data on this population remain limited. Clinicians need reliable expectations for post‐HoLEP PSA behaviour to avoid unnecessary anxiety, imaging or biopsy while still identifying the subset of patients at risk for progression.^7^, ^13^ Accordingly, the present study evaluates postoperative PSA kinetics after HoLEP in men with low‐risk PCa and compares these findings with those in men without PCa, thereby establishing a benchmark for expected PSA behaviour after HoLEP in low‐risk disease.

## 2 METHODS

### 2.1 Study design and patient population

We performed a retrospective review of a prospectively maintained HoLEP database at a tertiary academic centre, including cases from July 2017 through August 2025. Patients were stratified into two cohorts: (1) those with low‐risk PCa diagnosed either prior to or incidentally at the time of HoLEP and (2) those with no evidence of PCa on preoperative evaluation or postoperative histopathology.

**FIGURE 1:**
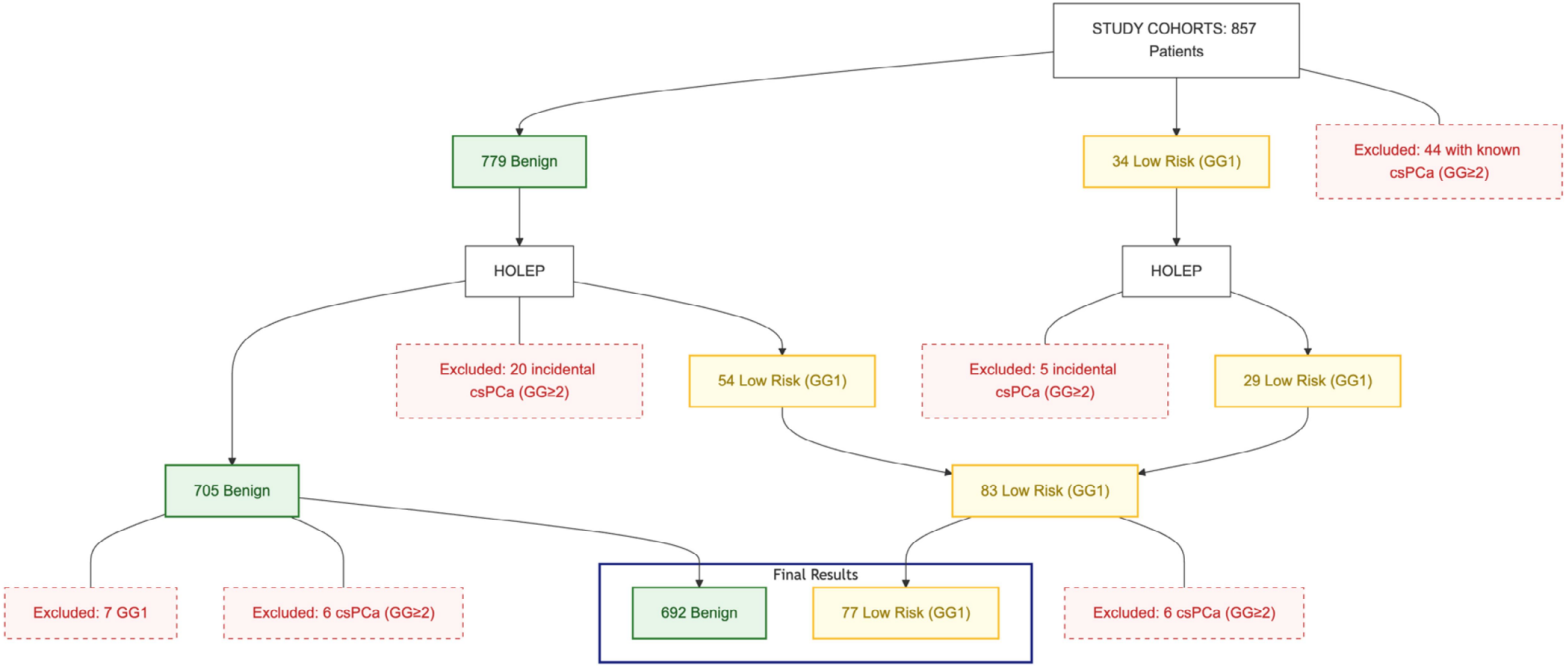
Study patient flow diagram. Flowchart illustrates patient selection, exclusion criteria and final grouping for analysis (*N* = 692 benign cohort; *N* = 77 low‐risk [GG1] cohort). csPCa, clinically significant prostate cancer; GG: grade group; HoLEP: holmium laser enucleation of the prostate.

Exclusion criteria included csPCa at any time point, prior PCa treatment and any upgrading from the benign cohort to low‐risk disease during follow‐up, to avoid cross‐contamination between groups that could confound PSA kinetics. The cohort selection process is summarized in Figure 1. Baseline characteristics, postoperative PSA kinetics and trends and oncologic outcomes were compared between groups. This study was approved by the institutional review board (IRB #20180511) and is reported in accordance with STROBE guidelines.^19^

### 2.2 Surgical technique, preoperative and perioperative management

Preoperative prostate size was determined using transabdominal or transrectal ultrasonography, computed tomography or magnetic resonance imaging performed within 12 months prior to surgery. Patients with elevated PSA underwent additional evaluation with adjunct testing (e.g., 4Kscore), multiparametric MRI and/or prostate biopsy, guided by shared decision‐making. Baseline variables were collected within 3 months prior to surgery.

All procedures were performed under general anaesthesia in the lithotomy position using an en bloc enucleation technique.^5^ A single surgeon performed all procedures with varying trainee involvement. Enucleation was performed using a 26‐French laser resectoscope (Karl Storz, Tuttlingen, Germany) and a 550‐μm laser fibre connected to a 100‐ or 120‐W holmium:YAG laser platform incorporating pulse modulation technology when available (Lumenis, Santa Clara, CA), with settings of 2 J and 30 Hz. Enucleated adenoma was morcellated intravesically using a mechanical morcellator (e.g., VersaCut™ [Lumenis, Santa Clara, CA] or Piranha™ [Richard Wolf, Vernon Hills, IL]).

All patients had first postoperative PSA at 3 month (±1 month) to look for nadir. Those with PSA < 1 were asked to repeat PSA annually and those with PSA > 1 ng/mL or any patients with rising PSA levels were given the option of further evaluation with shared decision‐making.

### 2.3 Data collection

Baseline variables included age, race, ethnicity, body mass index (BMI), prostate size, PSA, PSA density, family history of PCa, preoperative MRI status, PIRADS score and preoperative biopsy status and results. Intraoperative and postoperative variables included resected tissue weight; histopathology of the HoLEP specimen; PSA at 3 months, 1 year, 2 years, 3 years, 4 years and 5 years; postoperative MRI; postoperative PIRADS score; postoperative biopsy results; and duration of follow‐up.

### 2.4 Statistical analysis

Descriptive statistics were used to summarize baseline characteristics and postoperative outcomes. Normally distributed variables were summarized as mean with standard deviation (SD) and compared using *t*‐tests. Non‐normally distributed variables were summarized as median with interquartile range (IQR) and compared using the Wilcoxon test. Categorical variables were reported as frequencies and percentages and compared using Pearson's chi‐square test or Fisher's exact test, as appropriate. All statistical analyses were two‐sided, with *p* values <0.05 considered statistically significant. All statistical analyses were conducted in IBM SPSS Version 31.0.

## 3 RESULTS

A total of 769 patients met the inclusion criteria, comprising 692 patients in the benign cohort and 77 in the low‐risk cohort. Baseline characteristics were broadly comparable between groups. Median preoperative PSA was similar between groups (4.36 [2.20–7.96] vs. 4.33 [2.50–7.27] ng/mL, *p* = 0.740). Preoperative biopsy was more frequent in the low‐risk cohort (61.0% vs. 38.7%, *p* < 0.001) (Table 1).

**TABLE 1:** Baseline characteristics of benign and low‐risk cohorts.

Variable	Total (*N* = 769)	Benign (*N* = 692)	Low risk (GG1) (*N* = 77)	*p*
Age (years)	69.06 (8.52)	68.93 (8.50)	70.22 (8.62)	0.208
Race				0.744
White/Caucasian	645 (83.9%)	583 (84.2%)	62 (80.5%)	
Black	79 (10.3%)	70 (10.1%)	9 (11.7%)	
Asian	45 (5.8%)	39 (5.7%)	6 (7.8%)	
Ethnicity				0.105
Non‐Hispanic/Latino	350 (45.5%)	307 (44.4%)	43 (55.8%)	
Hispanic/Latino	389 (50.6%)	356 (51.4%)	33 (42.9%)	
BMI (kg/m^2^)	27.89 (4.48)	27.90 (4.45)	27.77 (4.71)	0.805
Prostate size (cc)	107.0 (75.0–158.0)	108.0 (75.0–160.0)	100.0 (81.0–135.5)	0.463
PSA (ng/mL)	4.34 (2.20–7.93)	4.36 (2.20–7.96)	4.33 (2.50–7.27)	0.740
PSA density (ng/mL^2^)	0.041 (0.024–0.071)	0.041 (0.024–0.071)	0.046 (0.026–0.075)	0.246
Family history of prostate cancer, *n* (%)	88 (11.4%)	79 (11.4%)	9 (11.7%)	1.000
MRI, *n* (%)	210 (27.3%)	183 (26.4%)	27 (35.1%)	0.107
PIRADS score				
PIRADS 1–2	6	6/183 (3.3%)	0	
PIRADS 3	36	31/183 (16.9%)	5/27 (18.5%)	
PIRADS 4	19	15/183 (8.2%)	4/27 (14.8%)	
PIRADS 5	4	3/183 (1.6%)	1/27 (3.7%)	
Biopsy, *n* (%)	315 (40.9%)	268 (38.7%)	47 (61.0%)	<0.001
Biopsy result				<0.001
Benign	290 (37.7%)	268 (100%)	22 (46.8%)	
Grade Group 1 disease	25 (3.3%)	0 (0.0%)	25 (53.2%)	

**FIGURE 2:**
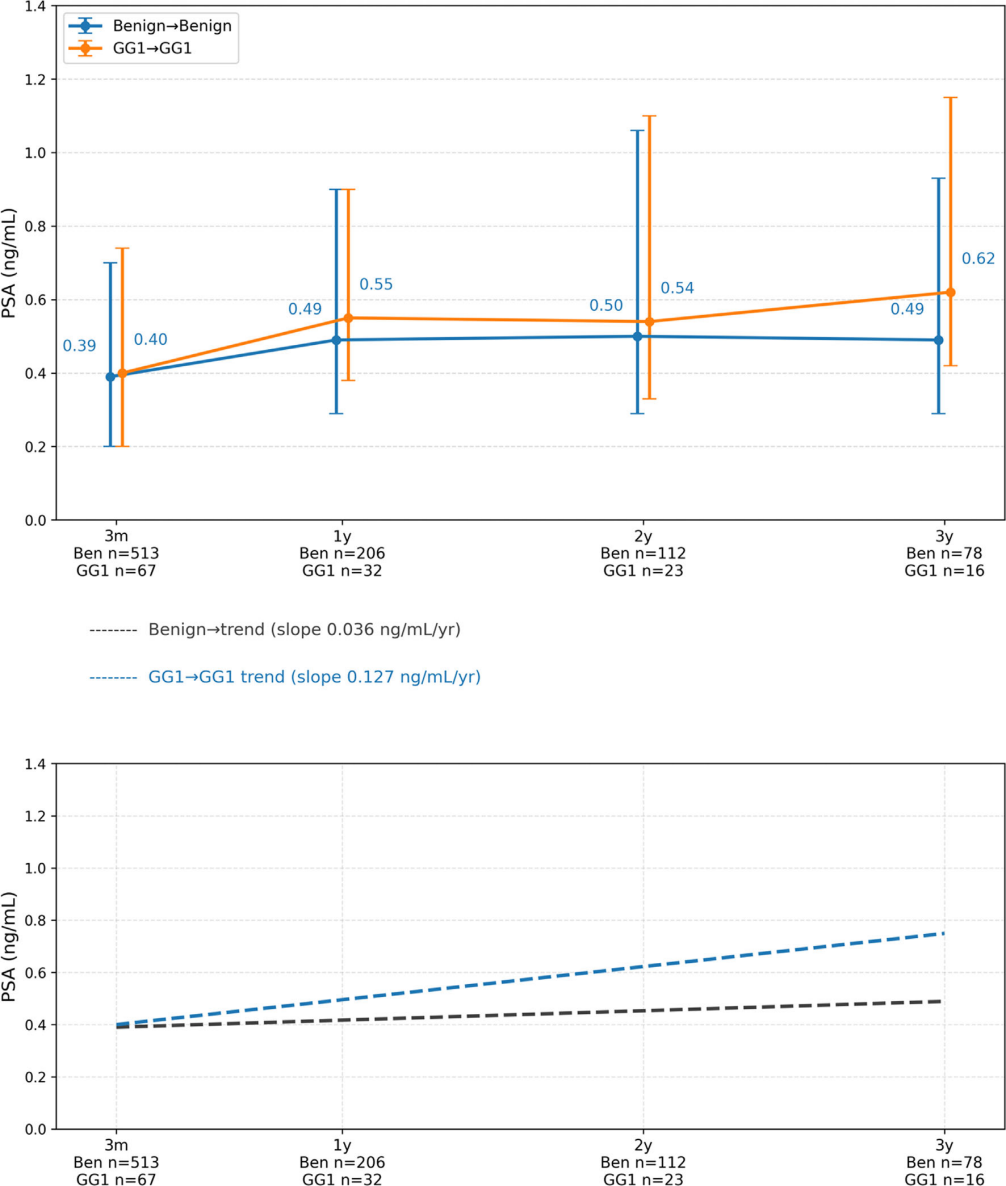
PSA trends of benign and low‐risk cohorts.

Early postoperative PSA dynamics were similar between cohorts. Both groups demonstrated a >90% decline in PSA following HoLEP. During follow‐up, PSA values remained low in both cohorts and did not differ significantly at selected timepoints. PSAV was slightly higher in the low‐risk cohort than in the benign cohort, though not statistically significant (0.127 [0.000–0.280] vs. 0.036 [−0.015–0.127] ng/mL/year, *p* = 0.259). PSA doubling time was shorter in the low‐risk cohort; however, this difference was not statistically significant (1.74 [1.19–3.64] vs. 2.40 [1.05–5.51] years, *p* = 0.297) (Table 2 and Figure 2).

**TABLE 2:** Intraoperative and postoperative outcomes of benign and low‐risk cohorts.

Variable	Total (*N* = 769)		Benign (*N* = 692)		Low risk (GG1) (*N* = 77)		*p*
Resected volume (g)	80.0 (47.2–116.0)		80.0 (47.5–116.0)		80.0 (44.0–110.0)		0.550
Histopathology of HoLEP specimen	709 (92.2%) benign 60 (7.8%) GG1		692 (100%) benign		17 (22.1%) benign^a^	60 (77.9%) GG1	<0.001
3 months PSA (ng/mL)	580	0.39 (0.20–0.70)	513	0.39 (0.20–0.70)	67	0.40 (0.20–0.74)	0.858
Postoperative PSA drop (PreOp PSA–3 months PSA)	567	3.97 (1.75–7.17)	500	3.93 (1.74–7.42)	67	4.00 (1.90–6.82)	0.934
Proportional PSA drop (%)	567	91.6 (81.3–95.8)%	500	91.6 (81.2–95.9)%	67	91.1 (83.0–95.5)%	0.953
1‐year PSA (ng/mL)	238	0.50 (0.30–0.90)	206	0.49 (0.29–0.90)	32	0.55 (0.38–0.90)	0.368
2‐year PSA (ng/mL)	135	0.50 (0.29–1.08)	112	0.50 (0.29–1.06)	23	0.54 (0.33–1.10)	0.602
3‐year PSA (ng/mL)	94	0.55 (0.29–0.96)	78	0.49 (0.29–0.93)	16	0.62 (0.42–1.15)	0.573
4‐year PSA (ng/mL)	60	0.49 (0.25–0.87)	52	0.49 (0.24–0.96)	8	0.46 (0.36–0.64)	0.845
5‐year PSA (ng/mL)	44	0.52 (0.29–0.86)	40	0.52 (0.29–0.93)	4	0.43 (0.24–0.63)	0.414
ΔPSA (PreOp – 3 years)	91	3.00 (1.50–6.11)	75	3.45 (1.50–6.60)	16	2.48 (1.65–2.80)	0.081
PSA velocity (3 months–3 years)	85	0.040 (−0.015–0.156)	70	0.036 (−0.015–0.127)	15	0.127 (0.000–0.280)	0.259
PSA doubling time (years)	188	2.20 (1.11–4.96)	158	2.40 (1.05–5.51)	30	1.74 (1.19–3.64)	0.297
Number of PSA ≥ 1 ng/mL, *n* (%)	138 (17.9%)		120 (17.3%)		18 (23.4%)		0.249
Number of PSA ≥ 1.5 ng/mL, *n* (%)	71 (9.2%)		64 (9.2%)		7 (9.1%)		1.000
Duration of follow‐up (months)	7.0 (3.1–22.0)		6.8 (3.0–22.1)		9.3 (3.6–21.4)		0.239

^a^ Patients who had known low‐risk disease preoperatively yielding benign histopathology included in low‐risk group.

During the post‐HoLEP surveillance period, 120 patients (17.3%) with BPH had PSA ≥ 1 ng/mL as compared to 18 patients (23.4%) with low‐risk PCa. Similar percentage of patients in both groups (9.2% in BPH and 9.1% in low‐risk PCa) were noted to have PSA ≥ 1.5 during post‐HoLEP PCa surveillance. A total of 58 prostate MRIs and 14 biopsies were performed during follow‐up based on shared decision‐making (Table 2).

## 4 DISCUSSION

HoLEP is known to produce a profound postoperative decline in PSA, often exceeding 80%, and establishes a new baseline.^4^, ^5^, ^6^ This marked ‘PSA reset’ complicates subsequent PCa screening and surveillance, as most conventional PSA thresholds were developed in surgery‐naive populations.^11^, ^12^ Prior studies have largely approached this issue in cancer‐enriched cohorts, where postoperative PSA levels and PSAV are typically higher in men with PCa, and postoperative cut‐offs of 1.0–1.2 ng/mL have been proposed as triggers for further evaluation in selected patients.^13^, ^15^, ^17^, ^18^ However, for men with low‐risk PCa managed after HoLEP, there remains little consensus regarding what degree of PSA increase over time should be considered expected versus concerning.^12^, ^20^ In the present study, PSA remained low in both cohorts, although the low‐risk group demonstrated a slightly greater upward drift over follow‐up. By quantifying PSA behaviour at prespecified timepoints and by PSAV, our findings assist in establishing a benchmark for expected PSA behaviour after HoLEP in low‐risk disease. The findings should provide a practical reference for postoperative counselling and surveillance in this population.

Baseline characteristics were broadly comparable between groups, including preoperative PSA and PSA density. This is consistent with the findings of Abedali et al., who also observed similar preoperative PSA values between benign and malignant cohorts.^17^ In contrast, Elmansy et al. and Lambert et al. reported higher preoperative PSA levels in patients with malignant pathology.^14^, ^18^ These differences may reflect variation in cohort composition, as prior malignant cohorts often included a broader range of disease severity, including csPCa, whereas our cancer cohort was limited to low‐risk disease. In the present study, median preoperative PSA and PSA density did not differ significantly between the benign and low‐risk cohorts, supporting the comparability of the two groups at baseline. In addition, 40.9% of the overall cohort had preoperative prostate biopsy, including 38.7% of patients in the benign group who had a negative biopsy before HoLEP. This suggests that a substantial proportion of men in our practice underwent evaluation for elevated PSA before surgery, reflecting a relatively intensive preoperative diagnostic approach.

Intraoperatively, the amount of resected tissue was similar between groups. Both cohorts demonstrated a median proportional PSA reduction greater than 90%, consistent with prior HoLEP series.^4^, ^5^, ^6^ Using the 3‐month postoperative PSA as the early nadir assessment, PSA values were 0.39 ng/mL in the benign cohort and 0.40 ng/mL in the low‐risk cohort. Reported post‐HoLEP PSA nadirs vary across the literature. Our findings fall within the lower range of previously reported post‐HoLEP nadir values, including those reported by Tinmouth et al., Martos et al., Elsaqa et al. and Lambert et al.^4^, ^5^, ^6^, ^18^ Although the central finding of a sharp postoperative PSA decline is consistent across studies, differences in nadir likely reflect variation in statistics, patient selection, PCa burden, residual gland size and surgical technique.

During long‐term follow‐up, PSA values remained broadly similar between cohorts through 5 years, supporting prior observations that HoLEP produces durable PSA suppression after tissue removal.^4^, ^5^, ^6^, ^18^ This pattern differs from biopsy‐selected or cancer‐enriched cohorts, in which postoperative PSA trajectories and PSAV may more clearly distinguish benign from malignant disease over time.^13^, ^15^, ^18^ In our study, differences were modest and did not reach statistical significance, suggesting a subtle numerical divergence rather than a pronounced separation in PSA trajectory. This likely reflects the composition of our study population, which was restricted to benign pathology and low‐risk disease and excluded patients with csPCa during postoperative follow‐up.

When summarized as post‐nadir PSAV, the low‐risk cohort demonstrated slightly greater upward drift than the benign cohort, although this difference was not statistically significant. This direction is consistent with prior work suggesting that higher postoperative PSAV after HoLEP or other outlet procedures may reflect oncologic signal.^13^, ^15^ However, the magnitude of the difference in our cohort was modest and should be interpreted cautiously, particularly given our exclusion of csPCa and upgrading events. These findings suggest that, in men with low‐risk PCa after HoLEP, modest PSA increases over time may occur without necessarily indicating clinically meaningful progression.

Our results also provide important context for interpreting commonly cited post‐HoLEP PSA thresholds. During postoperative surveillance, 138 patients had a PSA level of at least 1.0 ng/mL, and 71 had a PSA level of at least 1.5 ng/mL, demonstrating that values above these conventional thresholds can occur even in a relatively oncologically quiet cohort. This contrasts with biopsy‐selected series in which such thresholds were associated with a higher likelihood of csPCa. For example, Abedali et al. reported high rates of csPCa detection among post‐HoLEP patients selected for biopsy at PSA levels above 1.0 ng/mL, and Kimura et al. proposed a postoperative PSA cut‐off of approximately 1.2 ng/mL for cancer detection.^17^, ^21^ Our findings suggest that PSA thresholds derived from biopsy‐enriched or higher risk populations should be applied cautiously in men with benign pathology or low‐risk disease after HoLEP. Notably, postoperative surveillance in our cohort was also front loaded, with 58 postoperative MRIs and 14 postoperative biopsies performed, most clustering early after surgery. This pattern likely reflects early clinical uncertainty after PSA reset and reinforces the importance of contextual rather than reflexive interpretation of postoperative PSA elevations.

Overall, our data reinforce that HoLEP produces a profound PSA reset in patients with both benign pathology and low‐risk PCa. Postoperative PSA generally remains low in both groups, with only modest and non‐significant numerical divergence in long‐term trajectory. These findings provide practical reference values that may help support more consistent counselling and reduce unnecessary escalation in patients with stable benign or GG1 disease after HoLEP. At the same time, modest upward PSA drift should not automatically be considered reassuring or pathologic in isolation. Instead, surveillance should be individualized and interpreted in the broader context of preoperative evaluation, pathologic findings, PSA kinetics, imaging and shared decision‐making. Larger multi‐institutional studies with standardized surveillance protocols are needed to better define clinically meaningful PSA thresholds and trajectories in this setting.

This study should be interpreted in light of several limitations. First, its retrospective design introduces the potential for selection bias and unmeasured confounding. Second, postoperative surveillance with PSA testing, mpMRI and biopsy was not protocolized and instead reflected clinician judgement and shared decision‐making, which may have influenced detection patterns. Third, attrition over time was substantial, resulting in smaller analytic samples at later follow‐up points and limiting power for long‐term comparisons. Fourth, this was a single‐centre study that largely reflects the practice patterns of a single surgeon, which may limit generalizability to other settings. Finally, we excluded patients with a diagnosis of csPCa from our study because the number of such patients in the source population was limited; we were unable to evaluate PSAV or define PSA kinetic thresholds specifically for men with csPCa. Therefore, our findings should not be extrapolated to patients with csPCa after HoLEP. Future studies including larger cohorts with csPCa are needed to determine whether postoperative PSAV has different prognostic implications in this subgroup. Despite these limitations, our study provides a benchmark for expected PSA behaviour after HoLEP in patients with low‐risk PCa and without any PCa diagnosis.

## 5 CONCLUSION

HoLEP was associated with a marked postoperative PSA decline and persistently low PSA values during follow‐up in both benign and low‐risk PCa cohorts. Although patients with low‐risk disease demonstrated a slightly greater upward drift in PSA over time, postoperative trajectories remained broadly similar between groups. These findings suggest that modest PSA rises after HoLEP may be compatible with stable benign or GG1 disease, but their clinical significance remains uncertain. Given the nonprotocolized surveillance, attrition at later timepoints and limited long‐term sample size in the low‐risk cohort, these observations should be interpreted cautiously and require validation in larger studies.

## AUTHOR CONTRIBUTIONS

**Hasim Bakbak:** Conceptualization; data curation; investigation; methodology; visualization; writing—original draft; writing—review and editing. **Gurpremjit Singh:** Formal analysis; methodology; software; visualization; writing—review and editing. **Ahmad Abdelaziz:** Investigation; validation; writing—review and editing. **Juanita Velasquez Ospina:** Data curation; investigation; writing—review and editing. **Shlomo Resnik:** Data curation; investigation; writing—review and editing. **Connor Stephen Nee:** Data curation; investigation; writing—review and editing. **Archan Khandekar:** Investigation; methodology; validation; writing—review and editing. **Jonathan E. Katz:** Investigation; methodology; writing—review and editing. **Sanoj Punnen:** Methodology; validation; writing—review and editing. **Robert Marcovich:** Methodology; validation; writing—review and editing. **Mark L. Gonzalgo:** Methodology; validation; writing—review and editing. **Dipen J. Parekh:** Methodology; validation; writing—review and editing. **Hemendra Navinchandra Shah:** Conceptualization; investigation; methodology; project administration; supervision; validation; writing—original draft; writing—review and editing.

## CONFLICT OF INTEREST STATEMENT

The authors declare that no conflicts of interest exist.

## Data Availability

The data that support the findings of this study are available on request from the corresponding author. The data are not publicly available due to privacy or ethical restrictions.

## References

[1] Sandhu JS , Bixler BR , Dahm P , Goueli R , Kirkby E , Stoffel JT , et al. Management of lower urinary tract symptoms attributed to benign prostatic hyperplasia (BPH): AUA Guideline amendment 2023. J Urol. 2024;211(1):11–19. 10.1097/JU.0000000000003698 37706750

[2] Gravas S , Gacci M , Gratzke C , Herrmann TRW , Karavitakis M , Kyriazis I , et al. Summary paper on the 2023 European Association of Urology guidelines on the management of non‐neurogenic male lower urinary tract symptoms. Eur Urol. 2023;84(2):207–222. 10.1016/j.eururo.2023.04.008 37202311

[3] Das AK , Han TM , Hardacker TJ . Holmium laser enucleation of the prostate (HoLEP): size‐independent gold standard for surgical management of benign prostatic hyperplasia. Can J Urol. 2020;27(S3):44–50. 32876002

[4] Tinmouth WW , Habib E , Kim SC , Kuo RL , Paterson RF , Terry CL , et al. Change in serum prostate specific antigen concentration after holmium laser enucleation of the prostate: a marker for completeness of adenoma resection? J Endourol. 2005;19(5):550–554. 10.1089/end.2005.19.550 15989443

[5] Martos M , Katz JE , Parmar M , Jain A , Soodana‐Prakash N , Punnen S , et al. Impact of perioperative factors on nadir serum prostate‐specific antigen levels after holmium laser enucleation of prostate. BJUI Compass. 2021;2(3):202–210. 10.1002/bco2.68 35475131 PMC8988639

[6] Elsaqa M , Slade A , Lingeman J , Piroozi A , Wagner K , Jhavar S , et al. Holmium laser enucleation of prostate in patients with pre‐existing localized prostate cancer, dual center study. J Endourol. 2023;37(3):330–334. 10.1089/end.2022.0571 36463424

[7] Wei JT , Barocas DA , Carlsson S , Barocas D , Coakley F , Eggener S , et al. Early detection of prostate cancer: AUA/SUO guideline Part I. Prostate cancer screening. J Urol. 2023;210(1):46–53. 10.1097/JU.0000000000003491 37096582 PMC11060750

[8] Yilmaz M , Toprak T , Suarez‐Ibarrola R , Sigle A , Gratzke C , Miernik A . Incidental prostate cancer after holmium laser enucleation of the prostate—A narrative review. Andrologia. 2022;54(3):e14332. 10.1111/and.14332 34837229

[9] Porto JG , Blachman‐Braun R , Ajami T , Zarli M , Chen R , Furtado T , et al. Incidental prostate cancer after holmium laser enucleation of the prostate: critical analysis of independent risk factors and impact on surgical outcomes. BJUI Compass. 2023;5(3):374–381. 10.1002/bco2.306 38481670 PMC10927913

[10] Bâcle C , Seizilles De Mazancourt E , Abid N , Ruffion A , Rouvière O , Colombel M , et al. Impact of holmium laser enucleation of the prostate on active surveillance for prostate cancer in patients with lower urinary tract symptoms. Prostate. 2025;85(11):989–999. 10.1002/pros.24906 40275607

[11] Tosoian JJ , Mamawala M , Epstein JI , Landis P , Macura KJ , Simopoulos DN , et al. Active surveillance of grade group 1 prostate cancer: long‐term outcomes from a large prospective cohort. Eur Urol. 2020;77(6):675–682. 10.1016/j.eururo.2019.12.017 31918957

[12] Bhat A , Blachman‐Braun R , Herrmann TRW , Shah HN . Are all procedures for benign prostatic hyperplasia created equal? A systematic review on post‐procedural PSA dynamics and its correlation with relief of bladder outlet obstruction. World J Urol. 2022;40(4):889–905. 10.1007/s00345-021-03771-w 34212237

[13] Schober JP , Stensland KD , Moinzadeh A , Canes D , Mandeville J . Holmium laser enucleation of the prostate in men on active surveillance for prostate cancer with refractory lower urinary tract symptoms secondary to enlarged prostates. Prostate. 2023;83(1):39–43. 10.1002/pros.24433 36063405

[14] Elmansy HM , Elzayat EA , Sampalis JS , Elhilali MM . Prostatic‐specific antigen velocity after holmium laser enucleation of the prostate: possible predictor for the assessment of treatment effect durability for benign prostatic hyperplasia and detection of malignancy. Urology. 2009;74(5):1105–1110. 10.1016/j.urology.2009.06.039 19773035

[15] Helfand BT , Anderson CB , Fought A , Kim DY , Vyas A , McVary KT . Postoperative PSA and PSA velocity identify presence of prostate cancer after various surgical interventions for benign prostatic hyperplasia. Urology. 2009;74(1):177–183. 10.1016/j.urology.2008.10.062 19428074

[16] Nelson TJ , Javier‐DesLoges J , Deka R , Courtney PT , Nalawade V , Mell L , et al. Association of prostate‐specific antigen velocity with clinical progression among African American and non‐Hispanic White men treated for low‐risk prostate cancer with active surveillance. JAMA Netw Open. 2021;4(5):e219452. 10.1001/jamanetworkopen.2021.9452 33999164 PMC8129822

[17] Abedali ZA , Calaway AC , Large T , Lingeman JE , Mellon MJ , Boris RS . The role of prostate specific antigen monitoring after holmium laser enucleation of the prostate. J Urol. 2020;203(2):304–310. 10.1097/JU.0000000000000530 31487219

[18] Lambert E , Goossens M , Palagonia E , Vollemaere J , Mazzone E , Dell’Oglio P , et al. Changes in serum PSA after endoscopic enucleation of the prostate are predictive for the future diagnosis of prostate cancer. World J Urol. 2021;39(7):2621–2626. 10.1007/s00345-020-03444-0 32997261

[19] von Elm E , Altman DG , Egger M , Pocock SJ , Gøtzsche PC , Vandenbroucke JP , et al. The Strengthening the Reporting of Observational Studies in Epidemiology (STROBE) statement: guidelines for reporting observational studies. Ann Intern Med. 2007;147:573–577. 10.7326/0003-4819-147-8-200710160-00010 17938396

[20] Hutchison D , Peabody H , Kuperus JM , Humphrey JE , Ryan M , Moriarity A , et al. Management of prostate cancer after holmium laser enucleation of the prostate. Urol Oncol. 2021;39(5):297.e1–297.e8. 10.1016/j.urolonc.2020.11.003 33221258

[21] Kimura S , Katayama H , Ohara E , Aoki H , Shibuya R , Naganuma H , et al. Prostate‐specific antigen follow‐up and management for patients undergoing holmium laser enucleation of the prostate. Int J Urol. 2024;31(1):82–87. 10.1111/iju.15315 37803911

